# Publication Trends in Acupuncture Research: A 20-Year Bibliometric Analysis Based on PubMed

**DOI:** 10.1371/journal.pone.0168123

**Published:** 2016-12-14

**Authors:** Yan Ma, Ming Dong, Kehua Zhou, Carol Mita, Jianping Liu, Peter M. Wayne

**Affiliations:** 1 Sleep Center, Air Force General Hospital, PLA, Beijing, China; 2 Division of Interdisciplinary Medicine and Biotechnology, Beth Israel Deaconess Medical Center, Harvard Medical School, Boston, MA, United States of America; 3 IBM, Software Development Lab, Littleton, MA, United States of America; 4 Department of Health Care Studies & Daemen College Physical Therapy Wound Care Clinic Daemen College, Amherst, NY, United States of America; 5 Reference & Education Services, Countway Library of Medicine, Harvard Medical School, Boston, MA, United States of America; 6 Center for Evidence-Based Chinese Medicine, Beijing University of Chinese Medicine, Beijing, China; 7 Division of Preventive Medicine, Osher Center for Integrative Medicine, Brigham and Women's Hospital, Boston, MA, United States of America; Harvard Medical School, Boston, MA, United States of America; York University, CANADA

## Abstract

**Objective:**

Acupuncture has become popular and widely practiced in many countries around the world. Despite the large amount of acupuncture-related literature that has been published, broader trends in the prevalence and scope of acupuncture research remain underexplored. The current study quantitatively analyzes trends in acupuncture research publications in the past 20 years.

**Methods:**

A bibliometric approach was used to search PubMed for all acupuncture-related research articles including clinical and animal studies. Inclusion criteria were articles published between 1995 and 2014 with sufficient information for bibliometric analyses. Rates and patterns of acupuncture publication within the 20 year observational period were estimated, and compared with broader publication rates in biomedicine. Identified eligible publications were further analyzed with respect to study type/design, clinical condition addressed, country of origin, and journal impact factor.

**Results:**

A total of 13,320 acupuncture-related publications were identified using our search strategy and eligibility criteria. Regression analyses indicated an exponential growth in publications over the past two decades, with a mean annual growth rate of 10.7%. This compares to a mean annual growth rate of 4.5% in biomedicine. A striking trend was an observed increase in the proportion of randomized clinical trials (RCTs), from 7.4% in 1995 to 20.3% in 2014, exceeding the 4.5% proportional growth of RCTs in biomedicine. Over the 20 year period, pain was consistently the most common focus of acupuncture research (37.9% of publications). Other top rankings with respect to medical focus were arthritis, neoplasms/cancer, pregnancy or labor, mood disorders, stroke, nausea/vomiting, sleep, and paralysis/palsy. Acupuncture research was conducted in 60 countries, with the top 3 contributors being China (47.4%), United States (17.5%), and United Kingdom (8.2%). Retrieved articles were published mostly in complementary and alternative medicine (CAM) journals with impact factors ranging between 0.7 and 2.8 in the top 20 journals, followed by journals specializing in neuroscience, pain, anesthesia/analgesia, internal medicine and comprehensive fields.

**Conclusion:**

Acupuncture research has grown markedly in the past two decades, with a 2-fold higher growth rate than for biomedical research overall. Both the increases in the proportion of RCTs and the impact factor of journals support that the quality of published research has improved. While pain was a consistently dominant research focus, other topics gained more attention during this time period. These findings provide a context for analyzing strengths and gaps in the current state of acupuncture research, and for informing a comprehensive strategy for further advancing the field.

## 1. Introduction

The term “acupuncture” describes a family of procedures involving the stimulation of points on the body using a variety of techniques, with the goal of achieving therapeutic effects [1]. Acupuncture has been practiced widely in China, for thousands of years, as an integral part of traditional Chinese medicine (TCM). In recent decades, the practice of acupuncture has become increasingly prevalent world-wide, including being administered in leading academic medical centers [2–4]. In spite of this, the credibility and specific therapeutic applications for acupuncture are still highly debated in the medical community [1, 5–8]. The quantity, quality, origin, and nature of existing acupuncture research evidence play a central role in informing this debate.

To summarize the state of evidence in acupuncture efficacy and safety, inform clinical practice, and elucidate underlying mechanisms, a large number of systematic reviews and meta-analyses have been published in recent years. These reviews have addressed the evidence base for a range of conditions including back and neck pain [9–11], osteoarthritis [12, 13], nausea [14, 15], dental pain [16], menstrual cramps [17], and infertility [18, 19], among others. However, less is known at a broader scale regarding gross tends in research efforts. Since the quantity of academic publications within a given discipline, at least in part, reflects underlying strategies and priorities [20–22], it is valuable to objectively characterize publication trends and to compare them with other related disciplines [23–25]. Bibliometrics is a set of methods used to quantitatively analyze academic literature [21], and indicates productivity, quality (or "performance") and structural trends of researchers, organizations, or specific academic fields. As a simple form of big data analysis, it is useful in revealing historical development [20], quantifying existing trends [24], and predicting the future in a given research domain. Bibliometric methods have been applied to medical related topics [26–32] including complementary and alternative medicine [33–35]. The results of these findings can be used as context for analyzing broad scale strengths and gaps in the current state of evidence in a field, and for informing a comprehensive strategic plan for further advancing the field.

To date, little effort has been devoted to summarizing worldwide trends in acupuncture research publications. The present study aimed to quantitatively analyze trends in acupuncture publications over the past 20 years, including rates and patterns of acupuncture publication, study types/designs, clinical conditions addressed, countries of origin, and types and impact factors of journals.

## 2. Methods and Materials

This bibliometric study does not involve a research protocol requiring approval by an institutional review board or ethics committee.

### 2.1 Search tools

We used PubMed as a search engine to query the MEDLINE database. PubMed was chosen because it is a recognized primary tool for scholars in the medical field [36, 37], and it remains the optimal tool in biomedical electronic research [38]. PubMed was developed and is managed by the National Center for Biotechnology Information (NCBI), at the U.S. National Library of Medicine (NLM), located at the National Institutes of Health (NIH). A free resource, PubMed comprises over 24 million citations for biomedical literature [39], and provides daily access to millions of users. Almost 5 million queries are issued to PubMed each day by users around the world [37], who rely on frequent access to keep updated in biomedicine and make discoveries in their own fields [36].

PubMed/MEDLINE makes use of a controlled subject vocabulary, Medical Subject Heading (MeSH), which are assigned to records by subject specialist indexers who read the associated articles. Applying the MeSH vocabulary ensures that articles are uniformly and systemically indexed by research topic, regardless of the words used by the authors [40]. This makes it possible to analyze publication trends and changes in focus over time.

### 2.2 Search strategy

An online literature search was conducted using MeSH terms, title words, and author key words in PubMed/MEDLINE, with specified publication dates from 1995/01/01 to 2014/12/31. MeSH terms included Acupuncture; Acupuncture Therapy (not exploded); Acupuncture Analgesia; Acupuncture, Ear; Electroacupuncture; and Moxibustion. In addition, we searched acupuncture and electroacupuncture as title words and author key words for articles appearing in PubMed without MeSH for this time period. Manual checks were performed of retrieved articles for relevance. Inclusion criteria for identified citations were articles related to acupuncture research, including clinical trials, observational studies, basic human physiology studies, animal studies, theory and methods papers, systematic reviews/meta-analyses, case studies, and clinical practice guidelines. Exclusion criteria were 1) publication before 1995 or after 2014, and 2) publications indexed by MEDLINE but with insufficient data for screening, classification and analysis. Two authors (Y.M and M.D) extracted data independently. The total numbers of publications in each year were recorded during these targeted periods. Retrieval results were then further analyzed with respect to study types using PubMed filters. For the purpose of comparison, a blank search with specified publication dates from 1995/01/01 to 2014/12/31 was performed to retrieve general publications in biomedicine. To simplify analyses, articles retrieved over the 20 year inclusion period were grouped in 5-year increments. The annual growth rate (AGR) of publications was calculated as (Current Year Total–Previous Year Total)/Previous Year Total. A productivity index was calculated to compare the individual growth trend according to its baseline at year 1995, defined as (Current Year Total– 1995 Total)/1995 Total.

### 2.3 Journal analysis and impact factors

Journal Citation Reports (JCR) releases impact factor (IF), the most frequently used tool for evaluating journal performance within its field. Journal impact factor (JIF) is defined as the year’s average number of citations per paper published in a specific journal during the preceding 2 years [41]. In this study, JIF data were obtained from the JCR from Thomson Reuters. Articles were assigned the impact factor of their publishing journal in the year of publication, from which the JIF values of all publications were calculated.

### 2.4 Data analyses

Retrieved data was downloaded using PubMed's XML export function. Targeted information was extracted from original data (by Y.M and M.D), including MeSH terms, titles of articles, years of publication, article types, languages, journals and their ISO abbreviations, authors’ affiliations, country within which research took place. The XML file was processed using customized program (Automation of Internet Data Analysis), and all results were manually checked by the authors. The top 20 journals ranked by the total numbers of acupuncture-related publications over the entire observation period were identified. By defining the studied age groups in the filter, analyses on age-related publication trends were also performed. For demographic analyses of participants in clinical studies, age classes were defined as child (under 12 yr), adolescent (13–18 yr), adult (19–44 yr), middle aged (45–64 yr) and aged (65 yr and above).

### 2.5 Statistical analyses

SPSS 19.0 (IBM SPSS Statistics) was used for statistical analyses. Descriptive statistics were presented as means ± standard deviations. Differences in publication numbers across 5-year segments were assessed by one-way analysis of variance (ANOVA). Publication trends were assessed by exponential regression. A p-value <0.05 was considered statistically significant in ANOVA and regression models.

## 3. Results

### 3.1 Quantity of acupuncture publications

**Fig 1:**
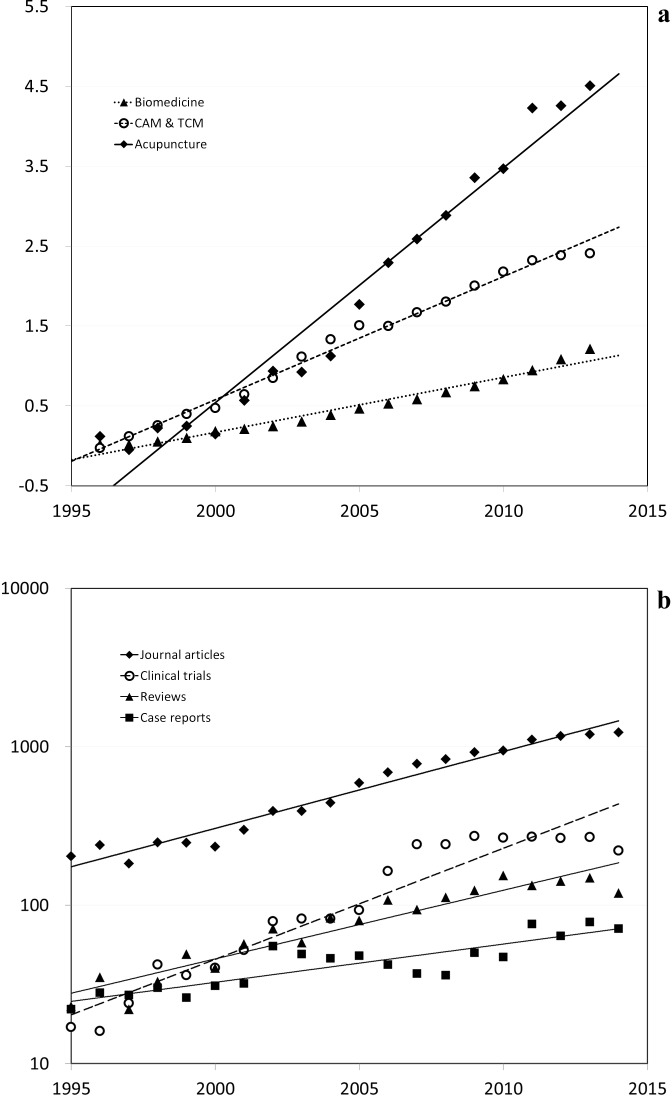
Growth in numbers of Pub Med research publications over the 20 year period, 1995–2014. **(a)** Biomedicine vs. complementary and alternative medicine (CAM) overall vs. acupuncture alone. The productivity index was calculated to compare the individual growth trend according to its baseline at year 1995, defined as (Current Year Total– 1995 Total)/1995 Total. **(b)** Exponential growing trends of some major types of acupuncture publications. The Y-axis is in log format.

A total of 13,320 citations were identified via PubMed as acupuncture research articles published during the targeted period of time (S1 File). Temporal analysis indicated an exponential growth in publications over the two decades evaluated, with a mean annual growth rate of 10.7%. This compares to a mean annual growth rate of 4.5% in biomedicine during the same period of time (Fig 1A). Thus, in biomedicine, the number of publications has doubled in the past two decades, while the number of acupuncture publications has increased more than four-fold.

### 3.2 Study types

Among all 13,320 publications, 12,339 (92.6%) were identified as journal articles (Table 1). Among journal articles, case reports comprised 7.3% of all articles (n = 895), non-randomized clinical studies 27.2% (n = 3351), randomized clinical trials 22.5% (n = 2771), narrative reviews 13.7% (n = 1686), systematic reviews 7.7% (n = 952) and meta-analyses 2.0% (n = 246). Among retrieved citations, 10,329 were human studies and 2,487 were animal studies (e.g. rats, mice, rabbits, etc.). Analyses showed that for each article type, the number of publications increased significantly in each 5-year period (Table 1). Among article types, randomized controlled trials have been increasing in numbers at the greatest rate, and along with other clinical studies, are now the most dominant acupuncture publication type (Fig 1B).

**Table 1:** Acupuncture publications in PubMed for 5-year intervals between 1995 and 2014.

Publication Categories	First 5 years (1995–1999)	Second 5 years (2000–2004)	Third 5 years (2005–2010)	Forth 5 years (2010–2014)	p	Mean AGR†
All publications	253.2 ± 29.9	398 ± 88.8	818.8 ± 137.1	1194.0 ± 102.8	<0.001	10.7%
Accessibility						
Full text available	89.2 ± 37.6	298.2 ± 107.2	496.6 ± 72.1	724.4 ± 62.9	<0.001	18.0%
Free full text available	16.2 ± 10.5	99.6 ± 56.3	153.8 ± 30.9	320.2 ± 86.4	<0.001	46.5%
Articles						
Journal article	224.2 ± 29.6	352.4 ± 84.2	762.4 ± 128.8	1128.8 ± 112.2	<0.001	11.4%
Clinical trial	42.2 ± 20.5	86.4 ± 27.2	237.8 ± 79.1	303.8 ± 26.2	<0.001	18.6%
RCT*	27.0 ± 11.6	67.0 ± 19.7	202.4 ± 73.0	257.8 ± 21.3	<0.001	19.5%
Review	32.4 ± 10.9	61.8 ± 16.2	103.6 ± 17.0	139.4 ± 13.9	<0.001	14.3%
Case report	26.6 ± 3.0	42.6 ± 10.6	42.6 ± 6.3	67.2 ± 12.5	<0.001	9.8%
Studied subjects						
Human	197.8 ± 38.3	333.6 ± 75.4	623.2 ± 164.9	911.2 ± 71.7	<0.001	11.6%
Animal	56.4±17.6	68.2±13.0	157.8±43.8	215.0±71.0	<0.001	8.2%

Results presented as mean±standard deviation of mean.

^†^AGR, annual growth rate, was calculated as (Current Year Total–Previous Year Total)/Previous Year Total

* RCT, randomized control trial.

### 3.3 Main health conditions addressed

The top 30 health conditions addressed among retrieved publications are listed in Table 2. Pain was the most commonly studied topic, accounting for approximately one-third of all identified publications. Other key areas of focus, listed in order of publication number, included cancer, arthritis, cerebrovascular diseases, pregnancy, inflammation, paralysis or palsy, mood disorders, and sleep disorders. A closer look at publication patterns comparing the first 10 years (1995–2004) with the last 10 years (2005–2014) reveals that the percentage of pain-related articles dropped slightly in the most recent 10 years, while other areas of research, namely arthritis, stroke, brain ischemia, fatigue, menopause, hot flashes, and irritable bowel syndrome, increased. Research evaluating acupuncture for pregnancy, mood disorders, sleep, and diabetes remained stable between the two 10-year time periods. Other less commonly studied health issues include edema, herpes zoster, temporomandibular joint dysfunction syndrome, hyperlipidemia, dyspepsia, tinnitus, vertigo, and dizziness.

**Table 2:** Overall ranking by topic and percentage of total acupuncture-related publications over the 20 year period, 1995–2014.

Rank	Focus areas by topic	Records (%)	1^st^ decade %	2^nd^ decade %	Rank	Focus areas by topic	Records (%)	1^st^ decade %	2^nd^ decade %
1	Pain	4601 (37.9)	40.5	37.0	7	Paralysis / Palsy	339 (2.8)	2.5	2.9
	Back Pain	401 (3.3)	3.9	3.1		Facial Paralysis & Bell Palsy	132 (1.1)	0.8	1.2
	Low Back Pain	317 (2.6)	3.0	2.5		Hemiplegia	68 (0.6)	0.7	0.5
	Headache	336 (2.8)	3.4	2.5		Cerebral Palsy	54 (0.4)	0.3	0.5
	Migraine	181 (1.5)	1.7	1.4	8	Brain Ischemia	339 (2.8)	2.2	3.0
	Neuralgia	175 (1.4)	1.8	1.3	9	Depression / Depressive Disorder	323 (2.7)	2.4	2.8
	Trigeminal Neuralgia	38 (0.3)	0.5	0.2	10	Allergy / Hypersensitivity	307 (2.5)	3.2	2.3
	Sciatica	30 (0.2)	0.5	0.2	11	Nausea / Vomiting	290 (2.4)	3.1	2.1
	Pelvic Pain	159 (1.3)	1.1	1.4	12	Sleep & Sleep Disorders	230 (1.9)	1.5	2.1
	Dysmenorrhea	102 (0.8)	0.6	0.9	13	Anxiety	179 (1.5)	1.2	1.6
	Arthralgia	150 (1.2)	1.0	1.3	14	Asthma	172 (1.4)	2.0	1.2
	Myofascial Pain Syndromes	122 (1.0)	1.0	1.0	15	Obesity	157 (1.3)	1.2	1.3
	Neck Pain	117 (1.0)	1.2	0.9	16	Diabetes Mellitus	131 (1.1)	1.1	1.1
	Shoulder Pain	81 (0.7)	0.6	0.7	17	Hypertension / Hypertensive	128 (1.1)	1.4	0.9
	Hyperalgesia	81 (0.7)	0.4	0.8	18	Fatigue	124 (1.0)	0.3	1.3
	Fibromyalgia	74 (0.6)	0.7	0.6	19	Dementia	120 (1.0)	0.7	1.1
	Facial Pain	56 (0.5)	0.8	0.3	20	Menopause	119 (1.0)	0.5	1.1
	Labor Pain	43 (0.4)	0.1	0.5	21	Hot flashes	105 (0.9)	0.3	1.1
2	Neoplasms	517 (4.3)	3.9	4.4	22	Infertility	105 (0.9)	0.5	1.0
	Breast Neoplasms	97 (0.8)	0.6	0.9	23	Parkinson	101 (0.8)	0.6	0.9
3	Arthritis	502 (4.1)	3.5	4.4	24	Spinal Cord Injuries	87 (0.7)	1.0	0.6
	Osteoarthritis	297 (2.4)	1.7	2.7	25	Irritable Bowel Syndrome	81 (0.7)	0.1	0.9
	Rheumatoid Arthritis	70 (0.6)	0.6	0.6	26	Rhinitis	80 (0.7)	0.6	0.7
4	Stroke	450 (3.7)	2.1	4.3	27	Epilepsy	68 (0.6)	0.7	0.5
	Cerebral Infarction	150 (1.2)	1.1	1.3	28	Smoking Cessation	62 (0.5)	0.8	0.4
5	Pregnancy	437 (3.6)	3.6	3.6	29	Tennis Elbow	47 (0.4)	0.6	0.3
	Labor, Obstetric	156 (1.3)	1.5	1.2	30	Diarrhea	46 (0.4)	0.4	0.4
6	Inflammation	361 (3.0)	2.8	3.0					

### 3.4 Countries from which publications originated

Our retrieved articles represented publications originating from 60 countries. The top 3 contributors of acupuncture-related publications were China (47.4%), United States (17.5%), and United Kingdom (8.2%), followed by South Korea (5.3%), Japan (2.8%), Germany (3.6%), Australia (1.8%), Canada (1.2%), Brazil (1.3%) and Italy (1.1%) (Table 3). Globally, total annual publication output increased from 230 in 1995 to 1261 in 2013. The top 3 countries in acupuncture publications remained relatively consistent over time, while Italy, South Korea, Germany and Australia exhibited higher annual growth rates in recent years.

**Table 3:** Top ranked countries with respect to numbers of acupuncture-related publications in the over the 20 year period, 1995–2014.

Rank in numbers of English language publications	Countries	Total number of publications in English (%)	Mean AGR^†^	Total number of publications overall l (%)	Overall rank in publications (any language)
1	China*	2330 (27.1%)	12.1%	6308 (47.4%)	1
2	United States	2316 (26.9%)	9.6%	2330 (17.5%)	2
3	United Kingdom^¤^	1096 (12.7%)	12.5%	1095 (8.2%)	3
4	South Korea	688 (8.0%)	23.0%	707 (5.3%)	4
5	Japan	326 (3.8%)	7.6%	369 (2.8%)	6
6	Germany	299 (3.5%)	19.4%	483 (3.6%)	5
7	Australia	242 (2.8%)	18.9%	242 (1.8%)	7
8	Canada	163 (1.9%)	17.6%	163 (1.2%)	9
9	Brazil	154 (1.8%)	12.9%	167 (1.3%)	8
10	Italy	128 (1.5%)	36.2%	142 (1.1%)	11
World Total	—	8598	10.7%	13320	—

^†^AGR, annual growth rate, was calculated as (Current Year Total–Previous Year Total)/Previous Year Total.

* Recorded numbers are from mainland China only.

^¤^ United Kingdom is calculated as the total of England, Scotland, Ireland and Wales.

### 3.5 Journal type and impact factors

**Fig 2:**
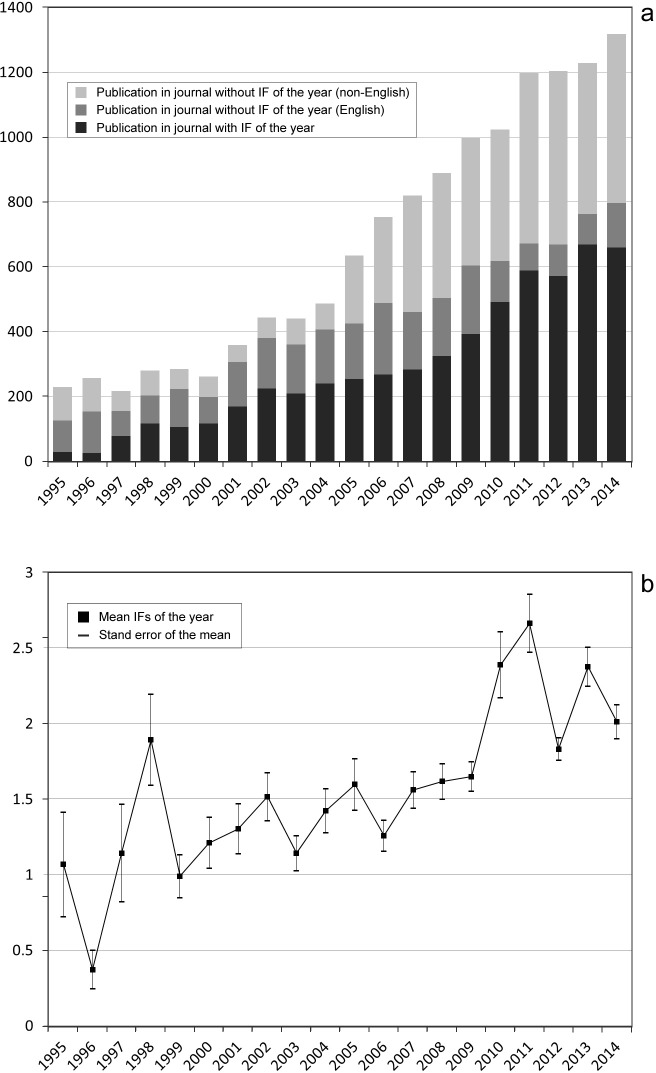
Impact factors characteristics for journals publishing acupuncture research articles between 1994 and 2014. **(a)** The number of publications in journals with and without impact factors at the time of publication. **(b)** The mean and stand error of impact factors of journals that acupuncture publications published each year. Note that non-JCR included non-English journals were not included in the calculation of means.

Name and category of journals as well as their impact factors, were recorded for all included acupuncture articles. Publications were retrieved from 1618 unique journals spanning 25 languages. Among English language journals, the top 20 titles with respect to total numbers of acupuncture publication are listed in Table 4. Twelve of the top 20 journals specialize in complementary and alternative medicine (CAM) or traditional Chinese medicine, with impact factors ranging between 0.7 and 2.8 (average = 1.5). The remaining 8 of the 20 most published in journals listed in Table 4 are biomedically oriented, and include periodicals with both broad (e.g. PLoS One, British Medical Journal) as well as specialized (e.g. Pain, Brain Research) medical themes. Impact factors for these journals were higher, ranging from 2.2 to 16.8 (average = 5.4). The number of journals publishing acupuncture papers has increased over the years, especially SCI-indexed journals and non-English journals (Fig 2A). When using the JIF to represent the impact of an article that was published in that year, an increasing trend over time in the average impact factors was observed.

**Table 4:** Top 20 journals that published acupuncture articles over the 20 year period, 1995–2014.

	Journal Titles	Abbreviations	Country of Publication	Records	%	2014 IF	5-Year IF
1	Journal of Traditional Chinese Medicine	J Tradit Chin Med	China	743	5.6%	0.716	0.749
2	Acupuncture in Medicine	Acupunct Med	England	619	4.6%	1.500	1.479
3	Journal of Alternative and Complementary Medicine	J Altern Complement Med	United States	492	3.7%	1.585	1.776
4	Evidence-Based Complementary and Alternative Medicine	Evid Based Complement Alternat Med	United States	379	2.8%	1.880	1.931
5	American Journal of Chinese medicine	Am J Chin Med	Singapore	252	1.9%	2.755	2.167
6	Acupuncture & Electro-Therapeutics Research	Acupunct Electrother Res	United States	172	1.3%	0.824	0.745
7	Journal of Acupuncture and Meridian Studies	J Acupunct Meridian Stud	South Korea	159	1.2%	N/A^†^	N/A^†^
8	Complementary Therapies in Medicine	Complement Ther Med	Scotland	154	1.2%	1.545	2.089
9	Neuroscience letters	Neurosci Lett	Ireland	124	0.9%	2.303	2.169
10	BMC Complementary and Alternative Medicine	BMC Complement Altern Med	England	120	0.9%	2.020	2.356
11	Chinese Journal of Integrative Medicine	Chin J Integr Med	China	104	0.8%	1.217	1.150
12	Cochrane Database of Systematic Reviews	Cochrane Database Syst Rev	England	104	0.8%	6.032	6.536
13	Pain	Pain	United States	77	0.6%	5.123	6.241
14	Trials	Trials	England	76	0.6%	1.731	2.162
15	PLoS One	PLoS One	United States	68	0.5%	3.234	3.702
16	Alternative Therapies in Health and Medicine	Altern Ther Health Med	United States	65	0.5%	1.243	1.526
17	British Medical Journal	BMJ	England	64	0.5%	17.445	16.814
18	Brain Research	Brain Res	Netherlands	62	0.5%	2.843	2.988
19	Explore-The Journal of Science and Healing	Explore (NY)	United States	55	0.4%	1.000	1.349
20	Anesthesia and Analgesia	Anesth Analg	United States	52	0.4%	3.472	2.454

^†^ N/A, not applicable.

Abbreviations: JCR, journal citation report; IF, impact factor.

### 3.6 Demographic characteristics of the clinical acupuncture studies

In order to analyze demographics of populations included in clinical acupuncture studies, we used PubMed customized filter settings, and chose five age groups across the lifespan. Articles that involved subjects from more than one age group were categorized in all corresponding age groups. Adult (19–44 yr) and middle-aged (45–64 yr) populations were studied the most with 4,249 and 3,749 articles respectively, followed by the aged (65 yr and above; 2,294), adolescents (13–18 yr; 1,106), and children under 12 years of age (619).

## 4. Discussion

Acupuncture is one of the most commonly used CAM therapies worldwide [42, 43]. In the Western world, its widespread use and growing integration into academic medical centers is due, in part, to early research demonstrating both potential clinical efficacy for a hand full of medical conditions, as well as plausible physiological mechanisms that explain clinical outcomes [6, 44]. However, despite significant effort, the evidence base for acupuncture for most medical conditions is still incomplete and debated [45, 46], and few attempts have been made to systematically map out trends in how research efforts to date have been focused.

Our bibliometric study of acupuncture research publications from 1995–2014 indicates an exponential growth in published evidence, with an average annual growth rate of 10.7%. This compares to a mean annual growth rate of 4.5% in biomedicine. The growth in acupuncture research publications parallels the increasing popularity of CAM therapies in general. Over the past decades, consumers’ demand for CAM, including acupuncture and related traditional Chinese medicine therapies, has increased [47, 48].

Paralleling the growth in absolute numbers of articles in the field of acupuncture research has been a trend towards more rigorous study designs and higher quality publications as reflected in journal impact factor scores. A striking trend our analyses revealed was an observed increase in the proportion of randomized clinical trials (RCTs), from 7.4% in 1995 to 20.3% in 2014. This increase in RCTs exceeded a more general background increase (4.5%) in the proportion of RCTs in biomedicine. These trends concur with more focused systematic reviews of acupuncture that report methodological quality of more recent trials has improved. This improvement, in part, may be linked to an increased demand by journals that they comply with Standards for Reporting Interventions in Clinical Trials of Acupuncture (STRICTA) guidelines [49].

Over the 20 year period we evaluated, pain was consistently the most common focus of acupuncture research (37.9% of publications). This focus includes both clinical and basic studies (3509 human studies versus 1092 animal studies), and encompasses a broad range of pain conditions. A number of systematic review and meta-analyses for chronic back pain [50–52] and headache [53–55] support the potential of acupuncture for treating pain-related symptoms, but the evidence of other pain conditions such as cancer-related pain [56, 57], fibromyalgia [58–60], and labor pain [61, 62] is mixed or negative. Regardless, pain management remains one of the primary indications for use of acupuncture by patients in the US and worldwide [4, 63]. Interestingly, compared to the overall growth in acupuncture research, the numbers of studies focused on pain did not increase between the first vs. second 10 years of our period of observation. Rather the overall increase in publications reflect a growing research interest in other conditions, including arthritis, neoplasms/cancer, pregnancy or labor, mood disorders, stroke, nausea/vomiting, sleep, and paralysis/palsy. Given clinical use of acupuncture for these and other conditions, future research should continue to target these areas.

Although our analyses revealed that acupuncture research was conducted in 60 countries, China, US, and UK were the top 3 contributors to the evidence base, contributing 47.4%, 17.5%, and 8.2%, respectively. These contributions remained fairly stable over time. However, a closer looks at contributions of higher quality studies (using RCTs as an indicator), reveals that higher quality studies were contributed mainly by the US and the UK in the first 10 years (1995–2004), while China did not have any RCTs in the record. However, in the second 10 years (2005–2014), the numbers of RCTs from China increased significantly more rapidly (yearly growth rate 56.2%) than the US (yearly growth rate 17.9%) and the UK (yearly growth rate 21.2%). Other countries, including Italy, South Korea, Germany and Australia, demonstrated a considerable trend in growth rates of publications, but absolute contributions were relatively small.

### Limitations

As with other publication analyses, some limitations are unavoidable. Exclusion of articles that were most recently published, not indexed by NLM, or not included in PubMed, make it impossible to trace all sources. In addition, although MEDLINE is the most authoritative international biomedical literature database, the number of medical journals from various countries is still limited. Other databases are also available for bibliometric studies, such as Web of Science, Scopus, Embase, PsychNet, and CINAHL. Another limitation is lack of granularity afforded by NLM filters, making it difficult to systematically classify publications as basic vs. clinical, or further classify basic research with respect to scope of research (e.g. immunology, neuroimaging, connective tissue physiology).

## Conclusion

Acupuncture research has grown markedly in the past two decades, with a 2-fold higher growth rate than biomedical research overall. Both the increase in the proportion of RCTs as well as the impact factor of journals published in support that the quality of published research has also improved. While pain was a consistent dominant research focus, other topics gained more attention during this time period. These findings provide a context for analyzing strengths and gaps in the current state of acupuncture research, and for informing a comprehensive strategy for further advancing the field.

## Supporting Information

S1 File Raw data file. Citations identified via PubMed during 1995–2014.
(7Z) Click here for additional data file.
